# A single dose of fluoxetine reduces neural limbic responses to anger in depressed adolescents

**DOI:** 10.1038/s41398-018-0332-2

**Published:** 2019-01-21

**Authors:** Liliana P. Capitão, Robert Chapman, Susannah E. Murphy, Christopher-James Harvey, Anthony James, Philip J. Cowen, Catherine J. Harmer

**Affiliations:** 1 Oxford University Department of Psychiatry, Oxford, England; 2 Oxford Health NHS Foundation Trust, Oxford, England

## Abstract

Depression in adolescence is frequently characterised by symptoms of irritability. Fluoxetine is the antidepressant with the most favourable benefit:risk ratio profile to treat adolescent depression, but the neural mechanisms underlying antidepressant drugs in the young brain are still poorly understood. Previous studies have characterised the neural effects of long-term fluoxetine treatment in depressed adolescents, but these are limited by concurrent mood changes and a lack of placebo control. There is also recent evidence suggesting that fluoxetine reduces the processing of anger in young healthy volunteers, which is consistent with its effect for the treatment of irritability in this age group, but this remains to be investigated in depressed adolescents. Here we assessed the effects of a single, first dose of 10 mg fluoxetine vs. placebo on neural response to anger cues using fMRI in a sample of adolescents with Major Depressive Disorder (MDD) who had been recently prescribed fluoxetine. As predicted, adolescents receiving fluoxetine showed reduced activity in response to angry facial expressions in the amygdala-hippocampal region relative to placebo. Activity in the dorsal anterior cingulate cortex (dACC) was also increased. No changes in symptoms were observed. These results demonstrate, for the first time in depressed adolescents, that fluoxetine has immediate neural effects on core components of the cortico-limbic circuitry prior to clinical changes in mood. The effect on anger is consistent with our previous work and could represent a key mechanism through which fluoxetine may act to alleviate irritability symptoms in adolescent depression.

## Introduction

Adolescence is a developmental period in which the risk of experiencing psychological disorders increases significantly. Depression is common during this age period, being associated with a high rate of recurrence and significant risk of suicide^1,2^. Clinically, adolescents with depression display the same symptoms as seen in adulthood, but there are some key differences: depressed youth often exhibit irritability rather than (or in addition to) low mood. This is reflected in the high rates of irritability reported in community and clinical youth samples with depression, varying between 30 and 85%^3–5^. For this reason, irritability is included as a cardinal symptom in the diagnosis of Major Depressive Disorder (MDD) among children and adolescents but not adults^6^. More recently, irritability has also been recognised as a core antecedent of depression in young people^7^, therefore playing an important etiological role in the development of depressive states. This association is further supported by evidence showing that depression and anger/irritability share overlapping genetic factors^8,9^.

Despite being a common disorder, the pharmacological treatments available to treat adolescent depression are limited, with only fluoxetine and escitalopram approved for use in the US and only fluoxetine in the UK. This problem is exacerbated by the fact that we still know very little about the neuropsychological mechanisms underlying antidepressant action in children and adolescents, which constrains the development of new drug treatments.

It is well established that depression in adolescence is associated with altered activity in the cortico-limbic circuitry underpinning affective processing. Indeed, it has been reported that depressed adolescents show overactive responses in the amygdala to negative stimuli, including to facial displays of anger^10^, as well as an increased tendency to detect angry faces^11,12^. The amygdala plays an important role in facilitating attention to salient stimuli and subsequent behaviour. Such anger bias has therefore been hypothesised to fuel irritable symptoms frequently seen in this population. Conversely, young people with depression show functional abnormalities in key areas subserving the development of emotional regulation, in particular the dorsal ACC (dACC)^13,14^. The dACC is a key neural substrate supporting the development of self-regulatory capabilities in adolescence, given its role in goal maintenance and integration of sensory and affective information to guide behaviour^15^. Functional impairments in this region are therefore thought to contribute to the difficulties experienced by depressed adolescents in self-regulating their negative emotions^13^.

Critically, there is evidence showing that 8-week treatment with antidepressants normalises amygdala^16^ and ACC^17^ responses to facial stimuli in depressed adolescents. Such effects are consistent with those believed to underlie treatment efficacy in adults^18^. However, studies in adolescents conducted to date are limited by the absence of a placebo control and the measurement of neural changes after relatively prolonged treatment schedules. Concurrent changes in symptoms at 8 weeks make it difficult to determine whether fluoxetine has a direct effect on cortico-limbic responsivity or whether the antidepressant-induced changes in activity are an indirect consequence of mood improvement and/or treatment expectations. The role on anger processing following antidepressant treatment has also not been explored despite the importance of this emotion for adolescent depression.

In adults, there is evidence that antidepressants have effects on emotional processing and related neural circuitry within hours of administration, and well before the therapeutic effects on mood emerge^19,20^. We have recently demonstrated that acute fluoxetine reduces the recognition of anger in young healthy volunteers aged 18–21 years old^21^, consistent with the hypothesis that anger processing may be core to the treatment action of fluoxetine in this age group. We hypothesise that this effect on perception by fluoxetine is associated with a reduction of neural activity in the amygdala, a key region involved in emotional processing and depression in adolescence^10^. To test this hypothesis, here we assessed the neural effects of a single dose of fluoxetine in depressed adolescents using a well-validated emotional faces paradigm, which included the presentation of angry faces. Adolescent patients with MDD were scanned using functional magnetic resonance imaging (fMRI) after taking their first dose of medication versus placebo. We predicted that fluoxetine would reduce neural activity in the amygdala in response to anger. Given the role of the dACC in adolescent depression^13,14^ and antidepressant effects^17^, we also expected to see an increase in activation in this region following fluoxetine administration.

## Methods

### Participants

Thirty-one adolescents with a primary DSM-IV diagnosis of MDD were recruited from Child and Adolescent Mental Health Services (CAMHS). CAMHS psychiatrists made the clinical decision to initiate fluoxetine treatment and determined that it was safe for the patient to wait 2–7 days before initiating treatment in order to be able to participate in the study. Exclusion criteria included: history of bipolar disorder or schizophrenia; substance abuse; current use of psychotropic/antidepressant medication, pregnancy and MRI incompatibility (presence of metal implants or claustrophobia).

This study was approved by the Southampton Research Ethics Committee (12/SC/0030). Participants aged 16 to 17 gave written informed consent. For participants younger than 16, written assent and consent were taken from the adolescent and their parent/guardian, respectively.

A formal sample size calculation was precluded, because no prior study had determined the acute effect of fluoxetine on brain activity in depressed adolescents. Hence, we estimated the likely effect size of acute antidepressant administration, and the likely minimum sample size, informed by two prior related studies. Our previous work showed that acute fluoxetine reduced facial recognition of anger, with an effect size of 0.81^21^. In a previous study with a similar fMRI paradigm, acute citalopram was found to reduce amygdala activation with an effect size of 1.19 (anatomically defined region of interest analysis)^22^. Informed by these data, an a priori sample size calculation for the current between-subjects design yielded *n* = 13 as the minimum sample size required to detect a reduction in amygdala fMRI signal of this magnitude (difference between two independent means: two tailed, alpha = 0.05, effect size = 1.19, power = 0.8).

### Procedures and measures

For a characterisation of the measures used in the screening, please refer to the Supplementary Information (SI).

Eligible patients were randomised to receive a single dose of either liquid fluoxetine (10 mg) or a matched placebo in a double-blind procedure. Placebo was peppermint syrup measured to the equivalent volume by a research psychiatrist not involved in the study^23^. Participants were asked to drink this mixture and then sat in a quiet room until the fMRI scan took place. The scan started 6 h after dosing, at a time where the plasma concentration of fluoxetine would be expected to be at its peak^24^.

Measures of state anxiety (STAI-C)^25^, mood (Visual Analogue Scale, adapted from Bond and Lader^26^) and side effects (Bodily Symptoms Checklist, adapted from Sinclair and colleagues^27^) were completed at three time points: before the drug/placebo administration, before the neuroimaging scan and immediately after.

After the testing session, participants were instructed to start fluoxetine treatment as prescribed by their treating Psychiatrist, who also managed their subsequent care.

### fMRI

#### Paradigm

The fMRI task^1^ included the rapid presentation of faces, to which participants had to respond by indicating the gender (male or female) as quickly and as accurately as possible via button press. The stimuli were colour photographs of angry, happy and fearful faces from the NimStim database^28^. Each trial began with the presentation of a fixation cross (2900 ms) followed by a face, which was presented in isolation for 100 ms. The task consisted of four 30 s blocks of each of the three conditions and there were ten faces presented per block. Between each block and at the start and end of the task, there was a 30 s baseline fixation cross, where participants were simply asked to stare at a fixation point. Blocks were presented in a fixed order (fearful, happy, angry repeated 4 times). This task has proved sensitive to the acute effects of antidepressants on neural processing^29^.

#### fMRI data acquisition and analysis

Details of fMRI data acquisition pre-processing and first-level analysis are provided in the SI.

Significant activations were identified using cluster-based thresholding of statistical images with a height threshold of *Z* > 2.3 and a (corrected) spatial extent threshold of *p* < 0.05^30^. At the whole-brain level, fearful and angry faces were contrasted with happy. Groups were contrasted with each other. Significant interactions from whole-brain analyses were further explored by extracting percentage BOLD signal change.

An anatomical ROI mask was created for the left and right amygdala hemispheres using the FSL Harvard-Oxford atlas. The dorsal and ventral ACC sub-regions were defined using a 8-mm sphere centred on the local maxima derived from Kujawa and colleagues^31^ and Beaver and colleagues^32^, respectively. The coordinates for the dorsal ACC (dACC) were *x* = –4, *y* = 30, *z* = 16, and for the ventral ACC (vACC) *x* = –18, *y* = 39, *z* = –12. All activations are reported using MNI coordinates. Group differences in the percentage signal change extracted from the amygdala and ACC masks were analysed using a mixed design (split-plot) ANOVA.

#### Statistical analysis of clinical and behavioural data

Demographic characteristics and baseline clinical measures were analysed using an independent sample *t* test or the non-parametric Mann–Whitney *U* test (when the assumption of normality was not ensured). Group differences between nominal variables were assessed using chi-square tests. A mixed design (split-plot) ANOVA was used to analyse group differences in self-report measures and behavioural performance in the emotional faces task. A *p* value lower than 0.05 was used to denote statistical significance. Partial eta squared (ηp2) are reported as a measure of effect size.

## Results

### Demographic and clinical characteristics

Demographic and baseline clinical characteristics are presented in Table 1. The final analysis consisted of 29 participants, as 3 participants did not complete the scan successfully. There were no significant group differences in age, gender distribution, ethnicity, IQ, family income or family composition, or any of the baseline clinical measures such as mean age of depression onset or number of comorbidities (all *p*s > 0.1). The groups were also comparable in terms of depression severity, trait anxiety, suicidal ideation or internalization/externalization symptoms (all *p*s > 0.09).

**Table 1:** Demographic and clinical characteristics

	Placebo (*n* = 15)	Fluoxetine (*n* = 14)
Socio-demographics		
Age (mean, SD)	15.67 (1.35)	16.00 (1.24)
Female:male ratio	12:3	10:4
Ethnicity (caucasian), *N* (%)	13 (86.67%)	14 (100%)
IQ (mean, SD)	114.67 (12.18)	111.57 (8.03)
Left:Right Handedness ratio	0:15	3:11
Family income level (mean, range)	3 (1–6)	3.5 (1–6)
Family composition (intact), *N* (%)	8 (53.33%)	7 (50.00%)
Clinical characteristics		
Duration of illness (months; mean, SD)	14.20 (8.65)	13.82 (11.12)
Age at onset of depression (mean, SD)	13.57 (2.22)	14.61 (1.44)
Number of MDD episodes (mean, range)	1.27 (1–2)	1.07 (1–2)
Psychotic features, *N* (%)	2 (13.33%)	3 (21.43%)
Low mood, *N* (%)	15 (100%)	13 (100%)
Irritability^a^, *N* (%)	7 (46.67%)	7 (50.00%)
Antidepressant naïve^b^, *N* (%)	12 (80.00%)	13 (92.86%)
Current psychological therapy/counselling, *N* (%)	9 (60.00%)	10 (71.43%)
Comorbid disorders^c^, *N* (%)		
None	8 (53.33%)	7 (50.00%)
Anxiety		
GAD	2 (13.33%)	0 (0.00%)
PTSD	2 (13.33%)	0 (0.00%)
OCD	1 (6.67%)	1 (7.14%)
Social phobia	1 (6.67%)	2 (14.29%)
Specific phobia	0 (0.00%)	3 (21.43%)
Panic disorder	2 (13.33%)	0 (0.00%)
Eating		
Anorexia	0 (0.00%)	1 (7.14%)
EDNOS	1 (6.67%)	1 (7.14%)
ASD		
Diagnosed	1 (6.67%)	1 (7.14%)
Probable	1 (6.67%)	1 (7.14%)
Fibromyalgia	1 (6.67%)	0 (0.00%)
Depression severity (mean, SD)		
CDI	32.79 (6.03)	29.77 (7.83)
CDRS-R	62.27 (8.64)	61.21 (13.34)
Trait anxiety (mean, SD)	51.27 (4.25)	48.15 (5.19)
State anxiety (mean, SD)	40.47 (4.73)	39.15 (4.41)
SIQ-Jr (mean, SD)	53.13 (24.99)	46.79 (21.41)
CBCL (mean, SD)		
Anxiety-depression	13.67 (4.48)	15.62 (2.96)
Aggression	9.50 (7.27)	9.92 (6.65)
Internalizing scores	28.25 (8.57)	29.31 (7.55)
Externalizing scores	11.92 (9.39)	13.62 (8.64)
Total scores	65.75 (21.88)	74.00 (21.40)

Family income per year was obtained using the following categories: 1 = Under £14,999; 2 = £15,000–£30,000; 3 = £30,000–£45,000; 4 = £45,000–£60,000; 5 = £60,000–£75,000; 6 = Above £75,000

*GAD* generalised anxiety disorder, *PTSD* post-traumatic stress disorder, *OCD* obsessive compulsive disorder, *EDNOS* eating disorder not otherwise specified, *ASD* asperger syndrome, *CDI* Children's Depression Inventory, *CDRS-R* Children's Depression Rating Scale, *SIQ-Jr* Suicidal Ideation Questionnaire - Junior, *CBCL* Child Behaviour Checklist

^a^As measured using the K-SADS-P (Schedule for Affective Disorders and Schizophrenia for School-Age Children-Present and Lifetime Version)

^b^All patients were antidepressant-naive apart from 4 (3 in the placebo group and 1 in the fluoxetine group). 3 of these patients had received treatment with fluoxetine in the past (due to depression) and another patient in the placebo group was taking amitriptyline for the treatment of fibromyalgia immediately before starting fluoxetine. This patient stopped taking amitriptyline for a period of 4 days before the testing session. This washout period was considered appropriate given that amitriptyline has a mean elimination half-life of 20 h (ranging from 9 to 46 h)

^c^According to DSM-IV criteria. Note: 4 patients in the placebo group had more than 1 comorbid disorder. 2 patients in the fluoxetine group had more than 1 comorbid disorder

### Subjective ratings

There was no significant effect of treatment on state anxiety or on any of the VAS scales (all *p*s > 0.7). Side effects were measured using a non-validated scale given the lack of suitable measures available to investigate acute antidepressant drug effects. Participants receiving fluoxetine reported a significantly lower number of bodily symptoms across all time points, i.e., even at baseline (F(1,24) = 6.874, *p* = 0.015, 4.82 vs. 2.97, ηp2 = 0.223).

### Behavioural performance

Accuracy in identifying the gender was high overall (>95%), therefore confirming that participants were engaged in the task. There were no group differences on accuracy or reaction times (all *p*s > 0.2) (Supplementary Table 2).

### **Main effect of task**

**Fig. 1:**
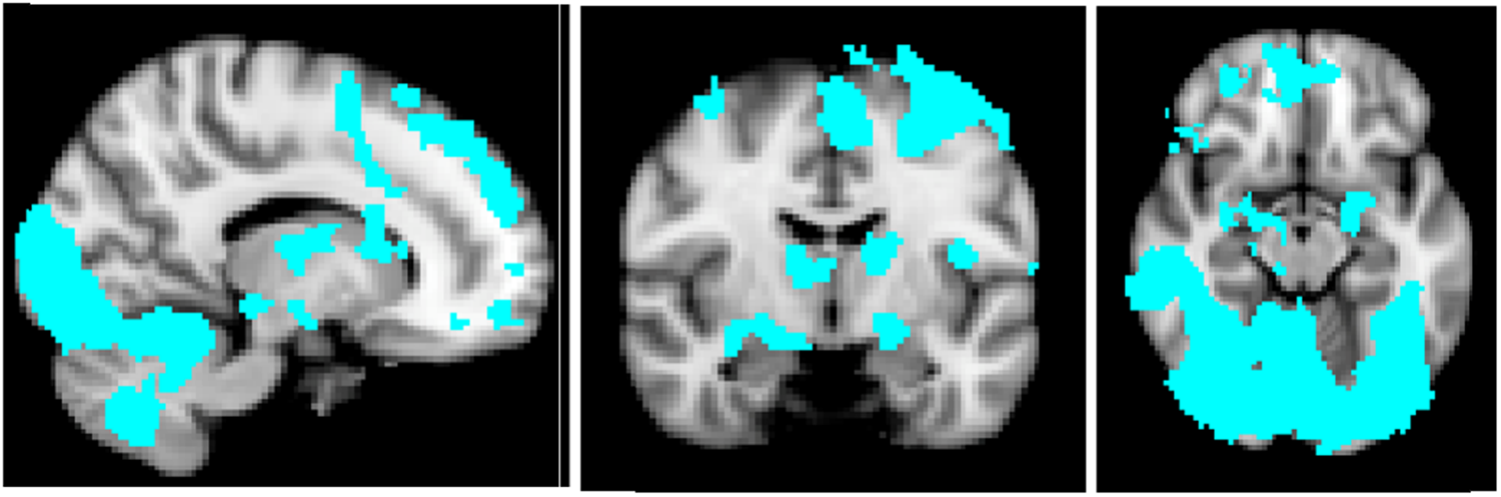
Whole-brain activation in response to anger vs. baseline (fixation) across groups. Sagittal, coronal and axial images depicting neural activation in response to anger vs. baseline (fixation) across groups. Images thresholded at Z > 2.3, p < 0.05, corrected

In order to determine if our task was engaging brain regions previously associated with angry facial stimuli, we compared neural activation in response to anger vs. baseline (fixation) across groups. Activity was observed in a network of areas that maps very closely to previous reports^33^, including the occipital fusiform gyrus, lateral occipital cortex, bilateral hippocampus, bilateral amygdala, cerebellum, thalamus, putamen, superior frontal gyrus, frontal orbital cortex, frontal pole, middle frontal gyrus, precentral and postcentral gyrus, insula and anterior cingulate gyrus (Fig. 1). These findings therefore confirm that this task engages brain regions that are part of a network relevant to anger.

### Effect of treatment

#### Whole brain analysis

**Fig. 2:**
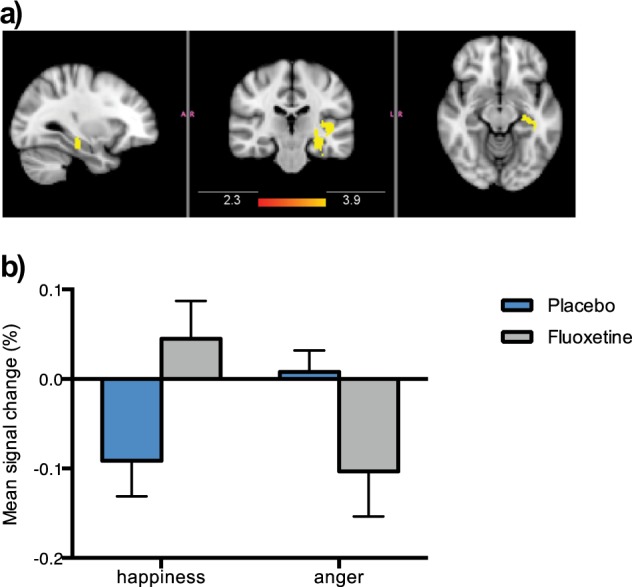
Whole-brain activation in response to angry vs. happy faces. **a** Sagittal, coronal and axial images depicting significantly reduced activation in the fluoxetine group for the anger vs. happiness contrast in a left temporolimbic cluster extending into the amygdala and hippocampus (peak voxels: *x* = −30, *y* = −24, *z* = −14; *Z* = 3.58; voxel size: 315). Images thresholded at *Z* > 2.3, *p* < 0.05, corrected. **b** BOLD percent signal change extracted from the significant left temporolimbic cluster in response to angry vs. happy faces. Error bars show standard error of the mean

A whole brain analysis (threshold *Z* > 2.3, *p* < 0.05, corrected) revealed reduced BOLD activation in the fluoxetine group relative to placebo in response to anger (vs. happiness) in a temporolimbic cluster in the left hemisphere, extending into the amygdala and hippocampus (*x* = −30, *y* = −24, *z* = −14; *Z* = 3.58, voxel size: 315). Patients who received fluoxetine showed relatively increased activation to happiness and reduced activation to anger, a pattern that is opposite to that seen in the depressed placebo group (Fig. 2). No group differences were seen in the contrast comparing fearful with happy faces.

#### Region of Interest (ROI) analysis

##### Amygdala

**Fig. 3:**
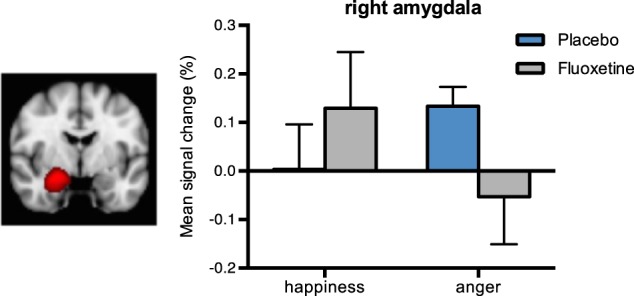
Mean percentage signal change from the anatomical mask in the amygdala. Mask from right hemisphere, created using Harvard-Oxford Atlas. Bars represent the mean percentage of signal change (%). Error bars show the standard error of the mean

There was a near significant interaction between emotion, hemisphere and group [F(1,27) = 4.034, *p* = 0.055, ηp2 = 1.30]. Similarly to that observed in the whole brain analysis, participants on fluoxetine (vs. placebo) showed a pattern of reduced activation in response to anger and increased activity in response to happiness (Fig. 3). Statistically, the group differences for anger were seen at a trend level (*p* = 0.082, ηp2 = 1.08), whilst no statistical differences were seen in response to happiness (*p* = 0.402). A similar analysis focused on the hippocampus revealed a similar pattern (see Supplementary Figure), suggesting that both regions may be involved in these effects of fluoxetine.

##### Anterior Cingulate Cortex (ACC)

**Fig. 4:**
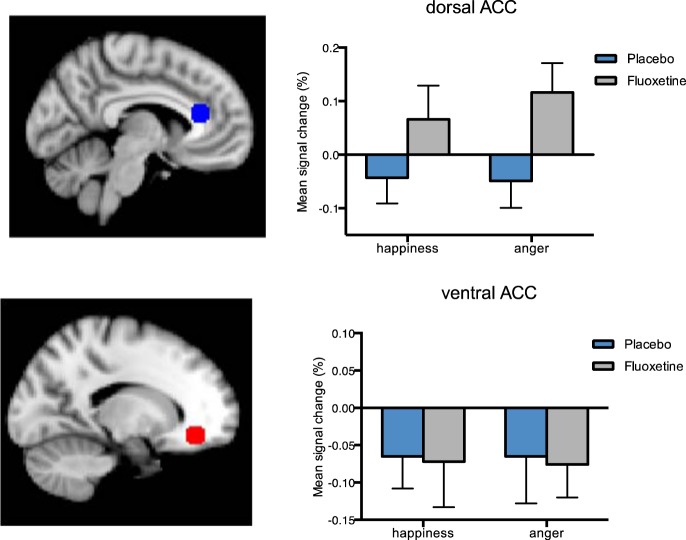
Mean percentage signal change from the anterior cingulate cortex (ACC). Top refers to dorsal ACC (dACC) and bottom to ventral ACC (vACC). Masks (8 mm spheres) were created based on the local maxima from Kujawa and colleagues^31^ and Beaver and colleagues:^32^ *x* = −4, *y* = 30, *z* = 16 (dACC), *x* = −18, *y* = 39, *z* = −12 (vACC). Sagittal view. Bars represent the mean percentage of signal change (%). Error bars show the standard error of the mean

There was a main effect of group when considering the dACC, with participants on fluoxetine showing increased activation in response to both happiness and anger [F(1,27) = 4.447, *p* = 0.044, ηp2 = 1.41]. No group differences were seen for the vACC [F(1,27) = 0.023, *p* = 0.880] (Fig. 4).

## Discussion

As predicted, a single dose of fluoxetine reduced neural activity to angry facial expressions relative to placebo in depressed adolescents, in a temporolimbic cluster that included both the amygdala and the hippocampus. Activity in the dACC was also increased. The demonstration that fluoxetine exerts direct and immediate effects on cortico-limbic activity in depressed adolescents, independently of changes in subjective symptoms, provides the first experimental evidence that, similarly to adults, antidepressants act early in treatment to modify brain regions implicated in adolescent depression. The effect on anger is consistent with our previous work and could therefore represent a key mechanism of action relevant to the treatment of adolescent depression, frequently characterised by irritability.

We have previously reported that acute fluoxetine reduces the accuracy to detect angry facial expression recognition in young healthy volunteers^21^. The present findings add to this work, by identifying a potential neural substrate for the early effects of fluoxetine on anger processing. Indeed, consistent with our hypothesis, participants on fluoxetine showed a reduced pattern of activity in response to anger (but not fear) vs. happiness in a temporolimbic cluster that extended into the amygdala. This finding was seen at a whole brain level, therefore surviving the stringent correction for multiple comparisons. The same pattern was also seen in the ROI analysis, although only as a trend. It is possible that the functionally-defined cluster observed in the whole brain corresponds to a more specific region of the amygdala, which detected relevant drug-related effects.

The amygdala has long been hypothesised to be a key site for antidepressant drug action, and the current study now provides direct support for an early effect of antidepressants on amygdala activity in adolescents. These results are consistent with previous adult studies showing that antidepressants have rapid effects on neural activity within hours of administration, and before patients start to notice any changes in subjective symptoms^19,20^. For instance, single doses of medications such as mirtazapine have been reported to reduce amygdala activity in response to fear vs. happiness in adults^29^. These changes are thought to represent a critical mechanism whereby antidepressants act to reduce the negative biases that characterise depressive states, whilst increasing the processing of positive information. Over time, these changes in emotional processing are believed to contribute to clinical improvements in mood, as the patient starts to perceive the world under a more positive light^19^. A reduction of amygdala activity following 8-week treatment with fluoxetine has been previously reported in a study with depressed adolescents^16^, but this is the first demonstration that such effects occur following just a single dose of fluoxetine and against a placebo control, therefore not being a consequence of symptom remission or treatment expectations.

The effect seen here on anger processing is consistent with our previous research and could therefore represent a key mechanism of fluoxetine for treating young people with depression, who frequently show irritability^3,4^. Indeed, depressed adolescents are particularly prone to detect angry faces^11,12^ and show hyperactive amygdala responses in response to this emotion^10^. Neural abnormalities in the amygdala, including to facial stimuli of anger, are also seen in other disorders characterised by irritability^34−37^. Fluoxetine may therefore act to reduce the salience of anger cues in the environment, an effect that could help reduce the symptoms of irritability over time. This hypothesis is supported by clinical evidence showing that fluoxetine is effective in treating anger in the context of adult depression with anger attacks^38,39^, intermittent explosive disorder^40,41^ and pre-menstrual disorder^42,43^. There are also preliminary data suggesting that selective serotonin reuptake inhibitors (SSRIs) – including fluoxetine - are beneficial in treating pathological symptoms of irritability/aggression across several paediatric disorders, not only depression, but also severe mood dysregulation and Attention Deficit Hyperactivity Disorder (ADHD) (see Kim and Boylan for a review)^44^. Future research should therefore examine the extent to which neural changes in response to anger are associated with behavioural changes in irritability following treatment with fluoxetine in a wide range of disorders. There is evidence from depressed adults showing that early SSRI-induced reductions in neural activity, including in the amygdala and ACC, to negative vs. positive faces are predictive of later therapeutic improvements^45^. It would be important for similar experimental medicine studies to be conducted in young people. Newer measures are also being developed to quantify irritability^46^, and these should form an integral part of future research into antidepressant effects in children and adolescents (see Vidal-Ribas for useful review)^9^.

Activity in the hippocampus was also reduced by fluoxetine in response to anger (vs. happiness). The hippocampus is commonly activated along with the amygdala in response to angry faces^47^ and following antidepressant treatment^29^, and lesions in the this structure have been reported to exert both anxiolytic and anti-aggression effects in rodents^48,49^. These findings are interesting in light of our previous work showing that acute fluoxetine not only reduced the recognition of anger in young healthy volunteers (aged 18 to 21) but also abolished the emotion-potentiated startle effect, thus showing effects consistent with an anxiolytic-like action^21^.

Participants receiving fluoxetine showed increased activity in the dACC in response to both anger and happiness. The dACC has been shown to respond to both positive and negative facial expressions^50^, a finding that could be attributed to its role in integrating affective information needed for self-regulation^51^. No significant group differences were seen in the vACC, therefore suggesting that these effects are specific to a region of the ACC involved in self-regulation and cognitive control^51,52^. There is considerable evidence showing that in adolescent depression the dACC displays a pattern of hypoactivity^14^ and other functional abnormalities^13,15^, hence the effects seen in this region could indicate that acute fluoxetine is normalising, to some extent, cognitive capabilities important for the regulation of emotions.

This study has limitations worth considering. Similar to populations in other adolescent depression studies^17^, many of the patients presented comorbid disorders, which could have influenced the results in unpredictable ways. This sample composition nonetheless reflects the characteristics of the target population, in which comorbidity is a norm rather than an exception^53^. Although we used a power calculation, our sample size was relatively small and future studies with a larger cohort of patients are important to replicate and further expand these findings. A range of ages was included in this study and there may be core differences in emotional processing during this key period of brain development. Future work may therefore wish to address adolescent stage as a moderator of antidepressant drug effects. Finally, the current study focused on the acute effects of fluoxetine on emotional neural processing, and hence neural changes were not correlated with symptomatic improvement that usually occurs over long time periods. Forthcoming studies should investigate whether these early effects are able to predict later symptomatic outcomes, in particular irritability.

Despite these limitations, the current study has important strengths. First, studies of neural effects of antidepressant drugs in young people are rare. Here we found that fluoxetine acts on neural correlates that have been previously implicated in adolescent depression and pathological irritability. Hence, this psychopharmacological approach could prove useful to screen new drug targets for adolescent depression and test the potential efficacy of fluoxetine in treating disorders in which anger and irritability are key symptoms. Second, the implementation, for the first time, of a single dose placebo-controlled design in unmedicated depressed adolescents allowed us to determine that the effects of fluoxetine on anger processing occurred prior to clinical changes in symptoms and independently of treatment expectations. Third, the drug effects in the amygdala-hippocampal region emerged in the whole brain analysis, therefore surviving a stringent correction for multiple comparisons.

## Conclusions

Fluoxetine has immediate neural effects in young people on core components of the cortico-limbic circuitry that could be relevant to the treatment of adolescent depression. The reduced activity in the amygdala-hippocampal region to angry facial expressions is consistent with our previous work, and could represent a key mechanism of action for subsequent improvement in symptoms of anger/irritability frequently seen in adolescent depression. Conversely, fluoxetine was shown to increase activity in the dACC, an important area involved in self-regulation, which has been implicated in the development of depression in this age group. Future studies should further explore the clinical implications of these effects, but it is hoped that these findings will assist the development of effective drug targets for adolescent depression, an area of research urgently needed.

## Disclaimer

The views expressed are those of the authors and not necessarily those of the NHS, the NIHR or the Department of Health.

## Supplementary information

Supplementary Methods and Results

## Footnotes

1 The emotional faces task was completed as part of a battery of fMRI tasks (others not reported here).
