# Supplementary material for: A single dose of fluoxetine reduces neural limbic responses to anger in depressed adolescents
Source: Transl Psychiatry. 2019 Jan 21;9:30. doi: 10.1038/s41398-018-0332-2 (PMC6341087; doi:10.1038/s41398-018-0332-2)
Supplement: Supplementary file 1 — Supplementary Methods and Results [file 41398_2018_332_MOESM1_ESM.docx]

**Supplementary Methods**

Procedures and measures

Psychiatric diagnoses were determined using the Schedule for Affective Disorders and Schizophrenia for School-Age Children-Present and Lifetime Version (K-SADS-P), administered either by a Child and Adolescent Psychiatrist (RB) or a Clinical Psychologist (LC). Depression severity was assessed using the Children’s Depression Inventory (CDI) and the Children’s Depression Rating Scale-Revised (CDRS-R). Participants were also administered the State-Trait Anxiety Inventory for Children (STAI-C) and the Suicidal Ideation Questionnaire-Junior version (SIQ-Jr). Parents/guardians completed the Child Behaviour Checklist (CBCL). IQ scores were determined using the 2-subscale version of the Wechsler Abbreviated Scale of Intelligence (WASI).

fMRI data analysis

Blood-oxygenation-level-dependent (BOLD) fMRI and T1-weighted anatomical images were acquired using a Siemens 3 Tesla TIM MAGNETOM Trio scanner, equipped with a 32-channel head matrix coil (Siemens, Erlangen, Germany) and located at the Oxford Centre for Clinical Magnetic Resonance Research (OCMR). Functional imaging consisted of 45 T2-weighted echoplanar imaging (EPI) slices (TR=3000ms, TE=30ms, matrix 64 x 64, slice thickness=3mm), 3mm^3^ voxels. An anatomical image (TR=2040ms, TE=4.7ms), 1mm^3^ voxels, was also acquired to allow later registration of the fMRI data into standard space.

fMRI data were pre-processed and analysed using FEAT (FMRI Expert Analysis Tool), version 5.09, part of FSL (FMRIB’s Software Library; [www.fmrib.ox.ac.uk/fsl](http://www.fmrib.ox.ac.uk/fsl)). Pre-processing involved a number of steps designed to reduce unwanted variability in the data and to improve the validity of statistical analyses: motion correction using FMRIB’s Linear Image Registration Tool (MCFLIRT; Jenkinson, Bannister, Brady, & Smith, 2002); deletion of non-brain tissue using the Brain Extraction Tool (BET; Smith, 2002); spatial smoothing with a Gaussian kernel of 5 mm full-width-half-maximum; grand-mean intensity normalisation of the entire 4D dataset by a single multiplicative factor; high pass temporal filtering (Gaussian-weighted least-squares straight line fitting, with sigma of 70s) and B0 unwarping using fieldmap phase and magnitude images for distortion correction. In addition, registration to high-resolution image and to a standard template [Montreal Neurological Institute (MNI)] was implemented using FNIRT nonlinear registration (Andersson, 2007).

In the first-level analysis, individual activation maps were computed using the general linear model with local autocorrelation correction. Three explanatory variables were modelled: “fear”, “happy” and “angry” faces. Temporal derivatives were included in the model as covariates of no interest to increase statistical sensitivity. Variables were modelled by convolving each block with a haemodynamic response function, using a variant of a gamma function (i.e. a normalisation of the probability density function of the gamma function) with a standard deviation of 3s and a mean lag of 6s.

In the second-level analysis, whole-brain individual data were combined at a group level (patients on placebo vs. fluoxetine) using a mixed-effects group cluster analysis across the whole brain corrected for multiple comparisons. Such a mixed-effects approach accounts for intra-subject variability and allows general population inferences to be drawn.

Absolute and relative motion values did not differ significantly between groups and no participant demonstrated significant movement (all participants revealed absolute motion ≤ 1mm). Relative motion was nonetheless added as a covariate of no interest.

At the whole-brain level, fearful and angry faces were contrasted with happy, resulting in the following model: 1) happiness vs. fixation; 2) fear vs. fixation; 3) anger vs. fixation; 4) happiness>fear; 5) happiness>anger; 6) fear>happiness; 7) anger>happiness. Groups were also contrasted with each other, resulting in the following comparisons: 1) placebo>fluoxetine; 2) fluoxetine>placebo. Significant interactions from whole-brain analyses were further explored by extracting percentage BOLD signal change for each emotion.

**Supplementary Results**

**Supplementary Table.** *Behavioural performance in the fMRI emotional faces task*

|  | **Placebo**  **(N=15),**  **mean ± SD** | **Fluoxetine**  **(N=14),**  **mean ± SD** |
| --- | --- | --- |
| **Accuracy (%)** |  |  |
| Happy | 95.83 (5.15) | 96.43 (3.06) |
| Fear | 95.33 (5.66) | 96.96 (3.13) |
| Anger | 97.00 (5.28) | 96.79 (4.85) |
| **Reaction Times (ms)** |  |  |
| Happy | 727.42 (147.18) | 680.59 (76.60) |
| Fear | 728.94 (139.75) | 684.69 (88.13) |
| Anger | 741.00 (140.12) | 683.29 (93.36) |

Region of Interest (ROI) analysis

When considering the hippocampus, there was a significant interaction between emotion and group [F(1,27)=4.731, p=0.039, ηp2=0.149], but follow-up pairwise comparisons failed to reveal any group differences for either happiness (p=0.503) or anger (p=0.222). However, the pattern of activation was the same as seen in the amygdala (reduced activation to anger and increased activation to happy, as shown in the figure below).


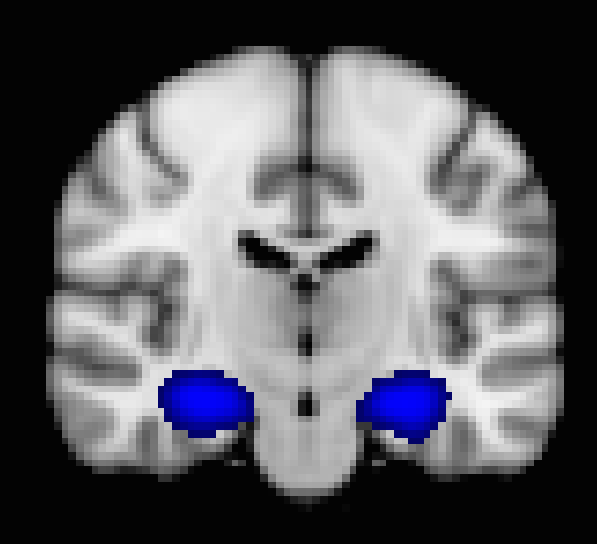


**Supplemental Figure.** **Mean percentage signal change from the anatomical mask in the hippocampus (both hemispheres combined), based in the Harvard-Oxford Atlas.** Bars represent the mean percentage of signal change (%). Error bars show the standard error of the mean.

**Supplementary References**

Andersson, M. Jenkinson and S. Smith (2007) Non-linear registration, aka Spatial normalisation. FMRIB technical report TR07JA2 from www.fmrib.ox.ac.uk/analysis/techrep.

Jenkinson M, Bannister P, Brady M, Smith S (2002). Improved optimization for the robust and accurate linear registration and motion correction of brain images. *NeuroImage* 17, 825–841.

Smith SM (2002). Fast robust automated brain extraction. *Human Brain Mapping* 17, 143–155.

Woolrich MW, Behrens TEJ, Beckmann CF, Jenkinson M, Smith SM (2004). Multilevel linear modelling for FMRI group analysis using Bayesian inference. *NeuroImage* 21, 1732–1747.

Woolrich MW, Ripley BD, Brady M, Smith SM (2001). Temporal autocorrelation in univariate linear modeling of FMRI data. *NeuroImage* 14, 1370–1386.
